# Differentiation in pyroptosis induction by *Burkholderia pseudomallei* and *Burkholderia thailandensis* in primary human monocytes, a possible cause of sepsis in acute melioidosis patients

**DOI:** 10.1371/journal.pntd.0012368

**Published:** 2024-07-23

**Authors:** Suphasuta Khongpraphan, Peeraya Ekchariyawat, Sucharat Sanongkiet, Chularat Luangjindarat, Stitaya Sirisinha, Marisa Ponpuak, Panuwat Midoeng, Matsayapan Pudla, Pongsak Utaisincharoen

**Affiliations:** 1 Department of Microbiology, Faculty of Science, Mahidol University, Bangkok, Thailand; 2 Department of Microbiology, Faculty of Public Health, Mahidol University, Bangkok, Thailand; 3 Department of Chemistry, Faculty of Science, Silpakorn University, Nakhon Pathom, Thailand; 4 Division of Pathology, Army Institute of Pathology, Phramongkutklao Hospital, Bangkok, Thailand; 5 Department of Oral Microbiology, Faculty of Dentistry, Mahidol University, Bangkok, Thailand; 6 Drug Discovery and Development Center, Office of Advanced Science and Technology, Thammasat University, Pathum Thani, Thailand; Instituto Butantan, BRAZIL

## Abstract

Melioidosis caused by *Burkholderia pseudomallei* is an infectious disease with a high mortality rate. In acute melioidosis, sepsis is a major cause of death among patients. Once the bacterium enters the bloodstream, immune system dysregulation ensues, leading to cytokine storms. In contrast to *B*. *pseudomallei*, a closely related but non-virulent strain *B*. *thailandensis* has rarely been reported to cause cytokine storms or death in patients. However, the mechanisms in which the virulent *B*. *pseudomallei* causes sepsis are not fully elucidated. It is well-documented that monocytes play an essential role in cytokine production in the bloodstream. The present study, therefore, determined whether there is a difference in the innate immune response to *B*. *pseudomallei* and *B*. *thailandensis* during infection of primary human monocytes and THP-1 monocytic cells by investigating pyroptosis, an inflammatory death pathway known to play a pivotal role in sepsis. Our results showed that although both bacterial species exhibited a similar ability to invade human monocytes, only *B*. *pseudomallei* can significantly increase the release of cytosolic enzyme lactate dehydrogenase (LDH) as well as the increases in caspase-1 and gasdermin D activations in both cell types. The results were consistent with the significant increase in IL-1β and IL-18 production, key cytokines involved in pyroptosis. Interestingly, there was no significant difference in other cytokine secretion, such as IL-1RA, IL-10, IL-12p70, IL-15, IL-8, and IL-23 in cells infected by both bacterial species. Furthermore, we also demonstrated that ROS production played a crucial role in controlling pyroptosis activation during *B*. *pseudomallei* infection in primary human monocytes. These findings suggested that pyroptosis induced by *B*. *pseudomallei* in the human monocytes may contribute to the pathogenesis of sepsis in acute melioidosis patients.

## Author summary

Acute melioidosis, caused by *B*. *pseudomallei* infection, is considered one of the lethal infectious diseases reported in Southeast Asia and Northern Australia. Infection with this bacterium can lead to a severe systemic inflammatory response or sepsis, which is a major cause of death. The mortality rate of acute melioidosis may reach up to 50%. In contrast, very rare cases were reported from patients infected with *B*. *thailandensis*, a non-virulence strain. Although it is well-documented that these two bacteria share genetic similarities and can exhibit similarities in survival and replication inside most murine and human cells, there is very limited information on how only *B*. *pseudomallei* can cause sepsis in melioidosis patients, while *B*. *thailandensis* dose not. We established an infection model using primary human monocytes to demonstrate that only *B*. *pseudomallei* can induce pyroptosis, causing the release of key cytokines that are involved in sepsis. This information may contribute to understanding the mechanisms underlying pathogenesis in acute melioidosis and may guide future treatments.

## Introduction

Melioidosis is an infectious disease caused by a pathogenic intracellular Gram-negative bacterium called *Burkholderia pseudomallei*, which is commonly found in soil and surface groundwater in tropical countries in Southeast Asia and Northern Australia [1]. The disease has a high mortality rate, and patients can exhibit a range of symptoms from acute to chronic and latent infections. Around 85% of melioidosis cases result in acute infection, which can cause severe sepsis (a life-threatening, systemic inflammatory and immune response that can cause organ dysfunction) and has a high case fatality rate reaching up to 50% [2]. The clinical manifestation in acute melioidosis patients is nonspecific, which can lead to misdiagnosis and result in delayed treatment. Furthermore, *B*. *pseudomallei* is naturally resistant to antibiotics and is not considered organisms that develop multidrug resistance [3,4] Therefore, understanding the host-microbe interaction is crucial for developing new therapeutic strategies [5]. In contrast to *B*. *pseudomallei*, *Burkholderia thailandensis* is generally considered a non-virulent species. Although *B*. *thailandensis* shares a high degree of genetic similarity with *B*. *pseudomallei* [6,7] there is no incidence of melioidosis caused by *B*. *thailandensis*. Among 1,200 melioidosis patients, no *B*. *thailandensis* isolates were identified, suggesting that only *B*. *pseudomallei* is virulent and causes the disease in humans [8]. The difference in virulence between these two bacterial species has also been investigated in mice, the mean 50% lethal dose (LD50) of *B*. *thailandensis* is 10^9^ CFU/mouse versus 182 CFU/mouse for *B*. *pseudomallei*, suggesting that *B*. *thailandensis* is less virulent [8, 9]. Therefore *B*. *thailandensis* has been widely used as a model organism for studying *Burkholderia* pathogenesis.

One of the major causes of acute melioidosis in patients is sepsis, which is defined as the systemic inflammatory response to infection [10]. In sepsis, the host immune system becomes hyperactivated and secrete a large quantity of inflammatory cytokines in the bloodstream, often referred to as cytokine storm. Interleukins are the most important cytokines released during infection, which include IL-1β, IL-18, IL-6, IL-12, and IL-17 [10,11]. In septic melioidosis, IL-1β and IL-18 are predominantly increased in the bloodstream of the patients [12,13]. These inflammatory cytokines are markers of the host innate immunological defense against invading pathogens and are produced during pyroptosis, a form of inflammatory programmed cell death pathway. In humans, this inflammatory cell death is activated by caspase-1/4/5 (caspase 1/11 in mice) and is characterized by cell swelling, membrane pore formation, and the release of cell contents and inflammatory cytokines such as IL-1β and IL-18 [14]. There are two pathways of pyroptosis: the caspase-1-dependent pathway and the caspase-1-independent pathway. The caspase-1-dependent pathway is activated when pathogen-associated molecular patterns (PAMPs) or damage-associated molecules pathogens (DAMPs) are recognized by inflammasome-initiating sensors such as NLRP3, NLRC4, AIM2, or Pyrin, which activate caspase-1. It was also demonstrated that mice lacking NLRP3 and NLRC4 exhibited more susceptibility to *B*. *pseudomallei* infection than the wild-type mice, implying that inflammasome also participates in resistance to melioidosis [12]. On the other hand, the caspase-1-independent pathway is activated when intracellular lipopolysaccharide (LPS) directly binds and activates caspase-11/4/5. Once activated, caspases cleave gasdermin-D in addition to the precursor cytokines pro-IL-1β and pro-IL-18 into their active forms. The cleaved N-terminus of gasdermin-D forms pores on the host cell membrane to mediate the release of cytoplasmic contents, including IL-1β and IL-18, and can also restrict *Burkholderia* spp. survival [14–17].

Even though several cell types in the bloodstream can release cytokines during infection, monocytes are considered to play a major role in cytokine secretion [18,19]. This blood cell type plays a crucial role in the innate immune system, particularly in the context of pyroptosis [20]. Monocytes travel throughout the bloodstream, detecting and responding to infections or other stimuli by producing inflammatory cytokines such as TNF-α, IL-1β, IL-6, IL-8, IL-10, and IL-12 [21]. These secreted cytokines can also activate other immune cells and trigger the production of additional cytokines, leading to a cytokine storm. Moreover, these cytokines provide an immunomodulating function by stimulating or inhibiting microbicidal activities [21,22]. However, an imbalance in the production of pro- and anti-inflammatory cytokines by these cells can lead to abnormal infection-induced immune responses.

Although *B*. *pseudomallei* has been extensively studied in several cell types, there is very limited information on how this bacterium affects human monocytes. Therefore, this study aimed to elucidate the difference in how primary human monocytes and human monocytic cell line (THP-1) respond to *B*. *pseudomallei* (virulent strain) and *B*. *thailandensis* (non-virulent strain). The results obtained may provide additional information explaining why only *B*. *pseudomallei* but not *B*. *thailandensis* can cause sepsis in patients.

## Materials and methods

### Ethics statement

The collection of buffy coat samples was carried out according to institutional guidelines and approved by Mahidol University Central Institutional Review Board (MU-CIRB): COA No. MU-MOU2022/062.2505.

### Bacterial strains and culture conditions

*B*. *pseudomallei* strain 1026b and *B*. *thailandensis* strain E264 were used in this study. Bacteria were cultured in Luria-Bertani (LB) broth at 37°C with agitation at 150 rpm. Overnight cultures were washed twice in phosphate-buffered saline (PBS) and adjusted to a desired concentration by measuring the optical density at 650 nm prior to infection experiments. For colony forming units (CFU) determination, bacteria were plated on tryptic soy agar and incubated at 37°C with aeration for 48 h.

### Human peripheral blood mononuclear cells and monocyte isolation

Peripheral blood mononuclear cells (PBMCs) were isolated from anonymized buffy coats provided by the Department of Medical Pathology, Phramongkutklao Hospital, using density gradient (Lymphoprep, Stemcells Technologies). CD14^+^ monocytes were isolated from PBMC by positive selection using a MACS system (Miltenyi Biotech, Bergisch Gladbach, Germany), according to the manufacturer’s protocol. After purification, 1 × 10^6^ cells/mL were cultured in 12-well flat-bottomed tissue culture plates (Nunc Inc., USA) in RPMI-1640 culture medium (Hyclone) supplemented with 5% heat-inactivated human serum (Sigma-Aldrich).

### Cell line and culture condition

THP-1 human monocytic cell line (ATCC-TIB-202) was used for comparison. The cells were grown in RPMI-1640 medium (Hyclone, Utah, USA) supplemented with 10% fetal bovine serum (Hyclone) and 1% L-glutamine (Gibco Labs, Grand Island, NY, USA) incubated at 37°C in 5% CO_2_ humidified. THP-1 cells were subcultured every 2 days by transfer to freshly RPMI-1640 medium culture.

### Monocyte infection, internalization, and intracellular survival assay

Primary human monocytes and THP-1 monocytic cells, 1.0 x 10^6^ cells/mL were infected with *B*. *pseudomallei* or *B*. *thailandensis* at a multiplicity of infection (MOI) of 10 for 1 h before cells were washed and incubated with kanamycin at a concentration of 250 μg/mL (Gibco) containing medium [23]. At indicated time points, the supernatant was collected for further assay. After washing steps, the monocytes were lyzed with 0.1% v/v Triton X-100. Serial dilutions of the lysate were prepared, and the bacteria present were enumerated using a drop plate technique on TSB agar. The total protein of infected cells was collected by 1X lysis buffer according to the manufacturer’s instructions.

### Cytotoxicity assay

Supernatants collected from bacteria-infected cells were subjected to a cytotoxicity test using the CytoTox 96 non-radioactive cytotoxicity assay (Promega, Wisconsin, USA) according to the manufacturer’s instructions. Cytotoxicity was measured by lactate dehydrogenase (LDH) releases as per the manufacturer’s instructions. The reaction was measured using a microplate reader (Ao Microplate reader, Azure biosystems Inc., Model AC3000) at 492 nm. A ratio was calculated by normalizing infected samples to their corresponding non-infected controls (set to 1) [24].

### Enzyme-linked immunosorbent assay (ELISA)

Cells were infected as described above, and cytokine concentration was determined in the culture supernatant to quantify human IL-1β and human IL-18 (R&D system) according to the manufacturer’s instructions. The reaction was measured using a microplate reader (Ao Microplate reader, Azure biosystems Inc., Model AC3000) at 450 nm.

### Immunoblotting

Equal amounts of protein were separated by 15% SDS-PAGE and transferred to a nitrocellulose membrane (Amersham Biosciences, UK) at a constant voltage of 90 volts for 2 h. The nitrocellulose was blocked using a 5% blocking solution (Roche, Switzerland) diluted in PBS at room temperature for 1 h. After blocking, the membrane was incubated with primary antibody against human caspase-1 (Cell Signalling; #2225) and cleaved-Gasdermin D (Cell Signalling, #36425) in a 5% blocking solution overnight at 4°C. As a loading control, all membranes were probed by anti-β-actin (Merck Millipore, Germany, MAB1501). The probed primary antibody membrane was washed with 0.1% Tween 20-PBS 3 times, 10 min each. Then, the membrane was incubated with horseradish peroxidase (HRP)-conjugated anti-rabbit IgG (R&D Systems, Minnesota, USA) for 1 h at room temperature. After 1 h of the secondary antibody probe, the membrane was washed with 0.1% tween 20 in PBS 4 times, 10 min each. The protein bands were visualized by incubating for 2 min with a chemiluminescence substrate (Roche Diagnostics, Basel, Switzerland) and exposed to hyperfilm (GE bioscience, USA).

### Flow cytometry analysis for ROS production

At the end of the infection, the primary human monocytes were washed twice with phosphate-buffered saline (PBS), 10 μM of 2’,7’-Dichloro-dihydro-fluorescein diacetate (DCFH-DA) (Sigma-Aldrich) were added and then incubated at 37°C for 30 min [25]. After that, cells were centrifuged at 4°C, 5000 rpm for 5 min, and washed with PBS twice. ROS production levels were then determined by using CytoFLEX flow cytometry (Beckman Coulter). The fluorescence of 10,000 cells was acquired and analyzed by the CytExpert program (Version 2.5).

### The multiplex immunobead-based cytokine assays

Culture supernatants from infected human monocytes were harvested at indicated time points. The multiplex immunobead-based cytokine assays were performed on a Custom Human ProcartaPlex Mix&Match 9-plex (ThermoFisher Scientific, USA) containing antibodies against human IL-1β, IL-1RA, IL-10, IL-12p70, IL-15, IL-18, and IL-23. In brief, 50 μl of cell culture supernatant was analyzed in duplicates. The assays were performed according to the manufacturer’s instructions, and the data acquisition was done using the MAGPIX program.

### Statistical analysis

All experiments were performed at least three independent times. The results were expressed as mean ± SEM. If conditions were to be compared, a paired t-test or one-way ANOVA followed by Tukey’s multiple comparisons were used. Graphing of data and statistical analyses were performed using the GraphPad Prism software. For the experiment with primary human monocytes, at least 3 different donors were used. Asterisks indicate statistically significant differences based on p-values: *p < 0.05, **p < 0.01, ***p < 0.001 and ****p < 0.0001.

The numerical data used in all figures are included in S1 Data.

## Results

### Intracellular survival of *Burkholderia* species inside human monocytes

**Fig 1:**
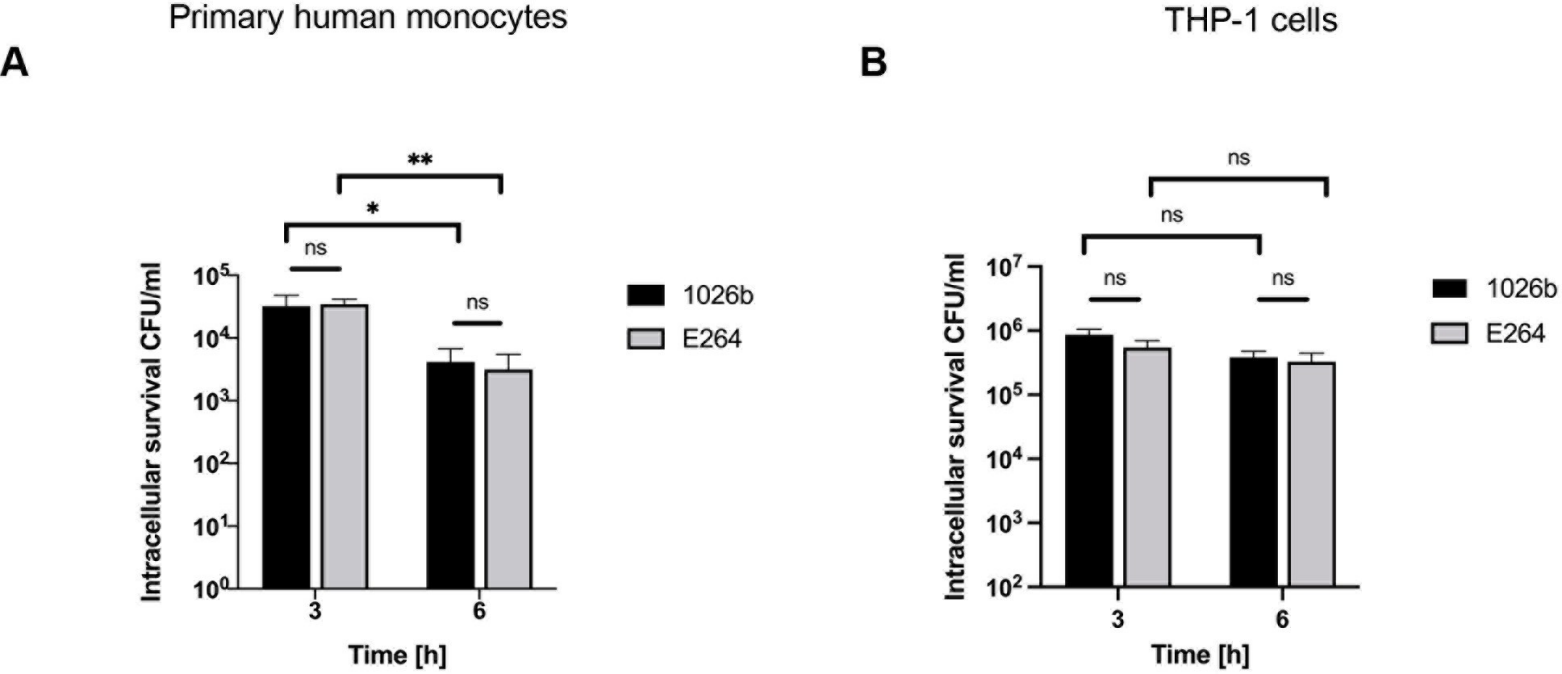
Intracellular survival of *B*. *pseudomallei* and *B*. *thailandensis* in primary human monocytes and THP-1 cells. Primary human monocytes **(A)** and THP-1 cells **(B)** were infected with *B*. *pseudomallei* or *B*. *thailandensis* at MOI of 10 and intracellular bacterial survival was determined at 3 h and 6 h post- infection. Data represent the results from at least 3 independent experiments from different donors. Data are represented as the mean ± SEM. (*p < 0.05 and **p < 0.01). ns (not significant).

*B*. *pseudomallei* is a facultative intracellular pathogen that can survive in both phagocytic and non-phagocytic cells [26,27]. In this study, we first investigated the ability of *B*. *pseudomallei* and its closely related species, *B*. *thailandensis*, to survive within primary human monocytes and THP-1 cells. We infected both cell types with these two *Burkholderia* species at an MOI of 10. At 3 h and 6 h post-infection, the intracellular survival of the bacteria was determined by measuring the CFU. Interestingly, both *Burkholderia* species showed similar intracellular survival in primary human monocytes and THP-1 cells (percentage uptake of both *Burkholderia* species was shown in S1 Table). Although both *Burkholderia* species are able to invade primary human monocytes and THP-1 cells, neither species was able to multiply inside the cells. The intracellular survival of both *Burkholderia* species significantly decreases only in primary human monocytes at the indicated time points [Fig 1A]. In contrast, the survival of both *Burkholderia* spp. slightly decreases, but not statistically significant in THP-1 cells [Fig 1B]. These results suggested that unlike other cell types, such as macrophages, *B*. *pseudomallei* and *B*. *thailandensis* can only be internalized but are unable to replicate inside the monocytes.

### *B*. *pseudomallei* induce pyroptosis activation in primary human monocytes and THP-1 cells

**Fig 2:**
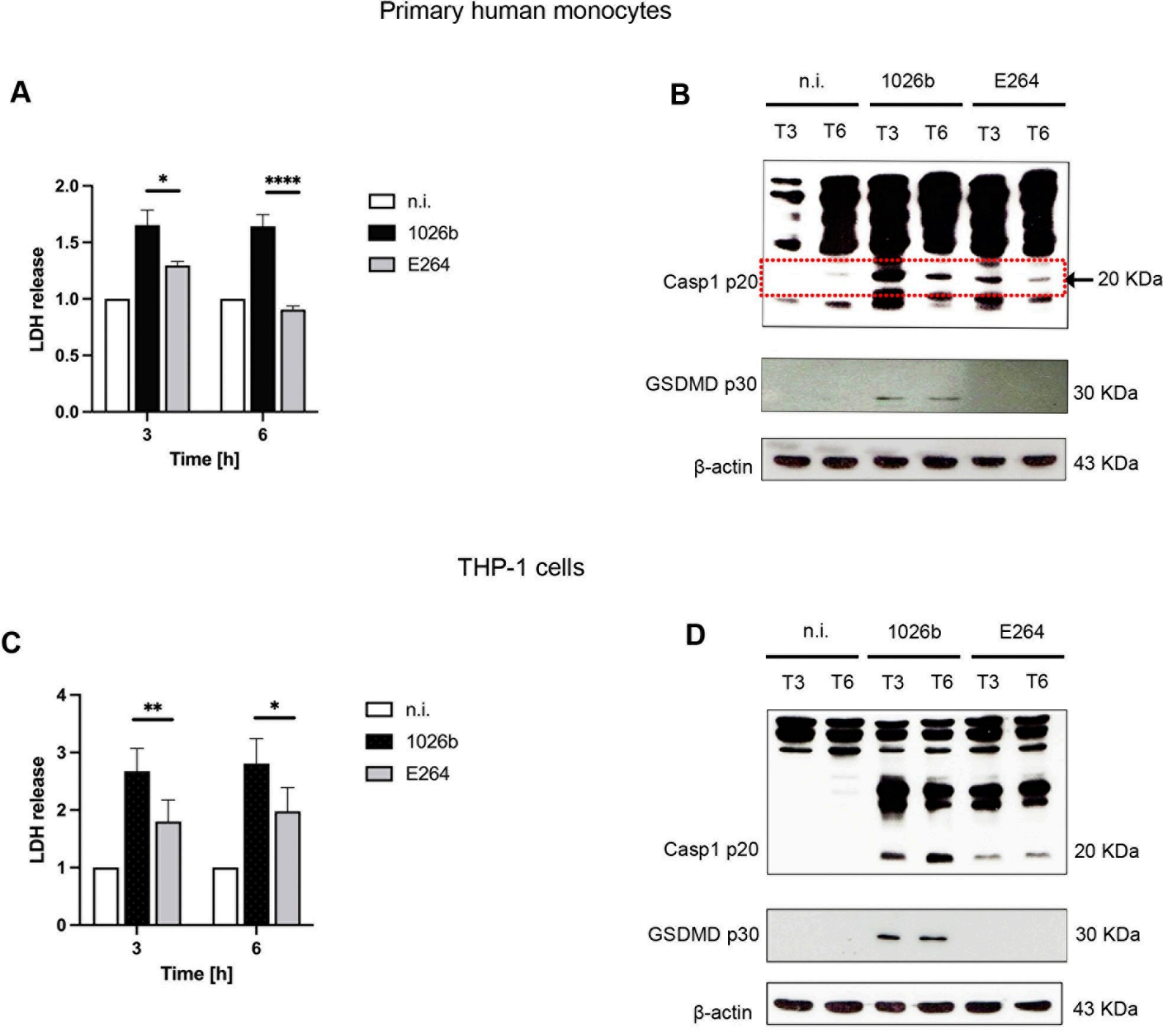
*B*. *pseudomallei* induces pyroptosis in primary human monocytes and THP-1 cells. Primary human monocytes and THP-1 cells were infected with *B*. *pseudomallei* or *B*. *thailandensis* at MOI of 10. At 3h and 6h post infection, the supernatant was collected and pyroptosis was determined by LDH assays **(A and C).** Immunoblotting was performed from the cell lysates (3 h and 6 h post-infection) for cleaved caspase-1 (casp1 p20) and Gasdermin-D (GSDMD p30) **(B and D).** The membrane was re-probed for β-actin. Data represent the results from at least 3 independent experiments from different donors. Data are represented as the mean ± SEM. (*p < 0.05, **p < 0.01 and ****p < 0.0001). n.i. (not infected).

Pyroptosis is an inflammatory programmed cell death pathway that responds to intracellular bacterial infections with unique characteristics: cells rupture and release pro-inflammatory cytokines such as IL-1β and IL-18 [15]. We further investigated whether both *B*. *pseudomallei* and *B*. *thailandensis* could induce pyroptosis in human monocytes in a similar fashion. As shown in Fig 2A, *B*. *pseudomallei* was able to stimulate higher levels of lactate dehydrogenase (LDH) release throughout the infection periods, suggesting that this bacterium caused pyroptosis in monocytes. These results were consistent with the activation of caspase-1 and GSDMD observed by immunoblotting [Fig 2B]. In contrast, the level of LDH release in *B*. *thailandensis*-infected primary human monocytes was similar to that seen in the uninfected control cells, suggesting that *B*. *thailandensis* failed to induce pyroptosis in primary human monocytes. These results were similarly observed in THP-1 cells, the level of LDH release seen in *B*. *pseudomallei*-infected cells was significantly higher than that of cells infected with *B*. *thailandensis* [Fig 2C]. These results are consistent with the activation of caspase-1 and GSDMD in these cells [Fig 2D]. Therefore, *B*. *pseudomallei* is significantly more potent in pyroptosis induction than the non-pathogenic strain *B*. *thailandensis* in primary human monocytes and THP-1 cells.

**Fig 3:**
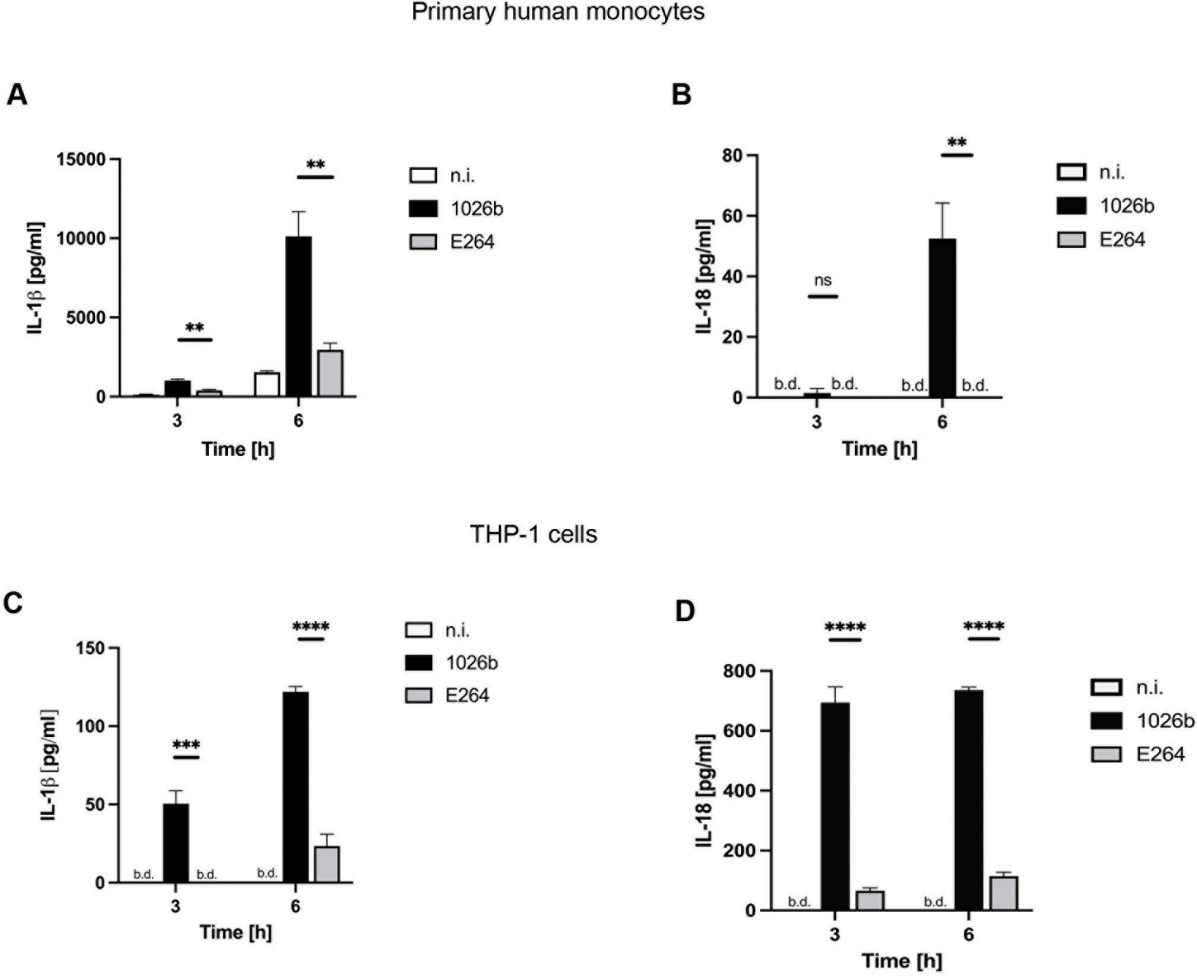
IL-1β and IL-18 production from primary human monocytes and THP-1 cells infected with *B*. *pseudomallei* or *B*. *thailandensis*. Primary human monocytes and THP-1 cells were infected with *B*. *pseudomallei* or *B*. *thailandensis* at MOI 10. At 3h and 6h post infection, the supernatant was collected and the levels of IL-1β **(A and C)** and IL-18 **(B and D)** production were quantified by ELISA assay. Data represent the results from at least 3 independent experiments from different donors. Data are represented as the mean ± SEM. (**p < 0.01, ***p < 0.001 and ****p < 0.0001). n.i. (not infected), b.d. (below detection). ns (not significant).

Since pyroptosis is directly related to pro-inflammatory cytokine secretion, particularly IL-1β and IL-18, we further investigated whether a higher level of pyroptosis observed in *B*. *pseudomallei*-infected cells compared to that of *B*. *thailandensis* was also consistent with the IL-1β and IL-18 secretion from both primary human monocytes and THP-1 cells. As expected, *B*. *pseudomallei*-infected cells significantly increased IL-1β and IL-18 secretion in both cell types in a time-dependent manner [Fig 3]. In contrast, *B*. *thailandensis*-infected cells failed to induce these pro-inflammatory cytokines. Altogether, our data suggested different modulations of pyroptosis in monocytes by *B*. *pseudomallei* and *B*. *thailandensis*.

### Cytokine secretion profiles of primary human monocytes infected *B*. *pseudomallei* and *B*. *thailandensis*

**Fig 4:**
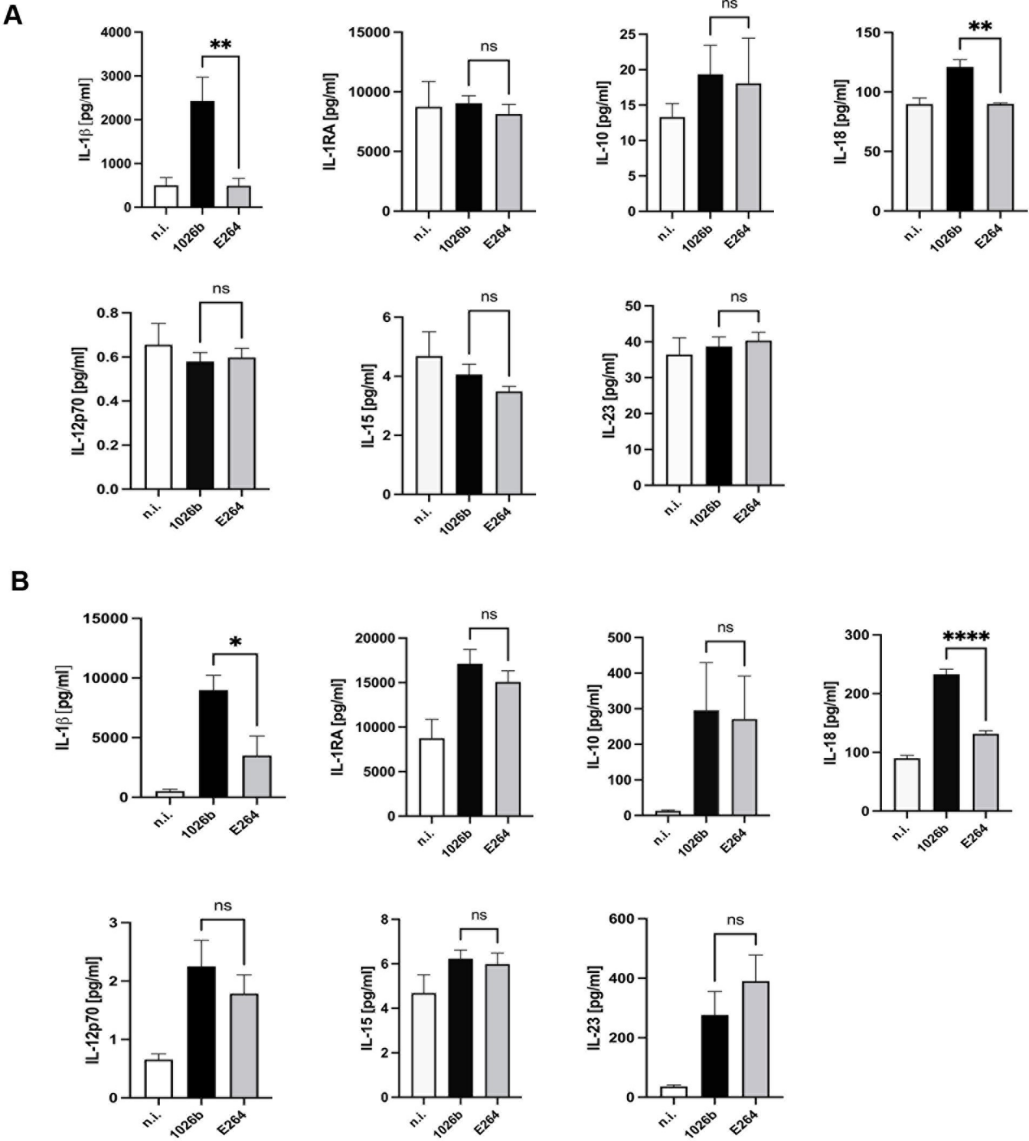
Comparing cytokine production profiles of *B*. *pseudomallei-* or *B*. *thailandensis-*infected primary human monocytes. Primary human monocytes were infected with *B*. *pseudomallei* or *B*. *thailandensis* at MOI of 10 for 3 and 6 h. At 3 h **(A)** and 6 h post-infection **(B)**, the levels of IL-1β, IL-1RA, IL-10, IL-18, IL-12p70, IL-15, and IL-23 were analyzed by the multiplex immunobead-based cytokine assays as described in Materials and Methods. Data represent the results from at least 3 independent experiments from different donors. Data are represented as the mean ± SEM. (*p < 0.05, **p < 0.01 and ****p < 0.0001). n.i. (not infected). ns (not significant).

Apart from examining the secretion of IL-1β and IL-18, we also investigated the differences in cytokine secretion profiles between *B*. *pseudomallei*- and *B*. *thailandensis-*infected primary human monocytes. Surprisingly, the results showed that only IL-1β and IL-18 levels were significantly higher in cells infected with *B*. *pseudomallei* than those infected with *B*. *thailandensis* at 3 and 6 h post-infection [Fig 4A and 4B]. On the other hand, the levels of IL-1RA, IL-10, IL-12p70, IL-15, and IL-23 were not significantly different between the cells infected with the two bacterial species throughout the infection periods. This observation implied that IL-1β and IL-18 may partly participate in the pathogenesis of sepsis in *B*. *pseudomallei* infection.

### *B*. *pseudomallei*, but not *B*. *thailandensis* produces reactive oxygen species (ROS)

**Fig 5:**
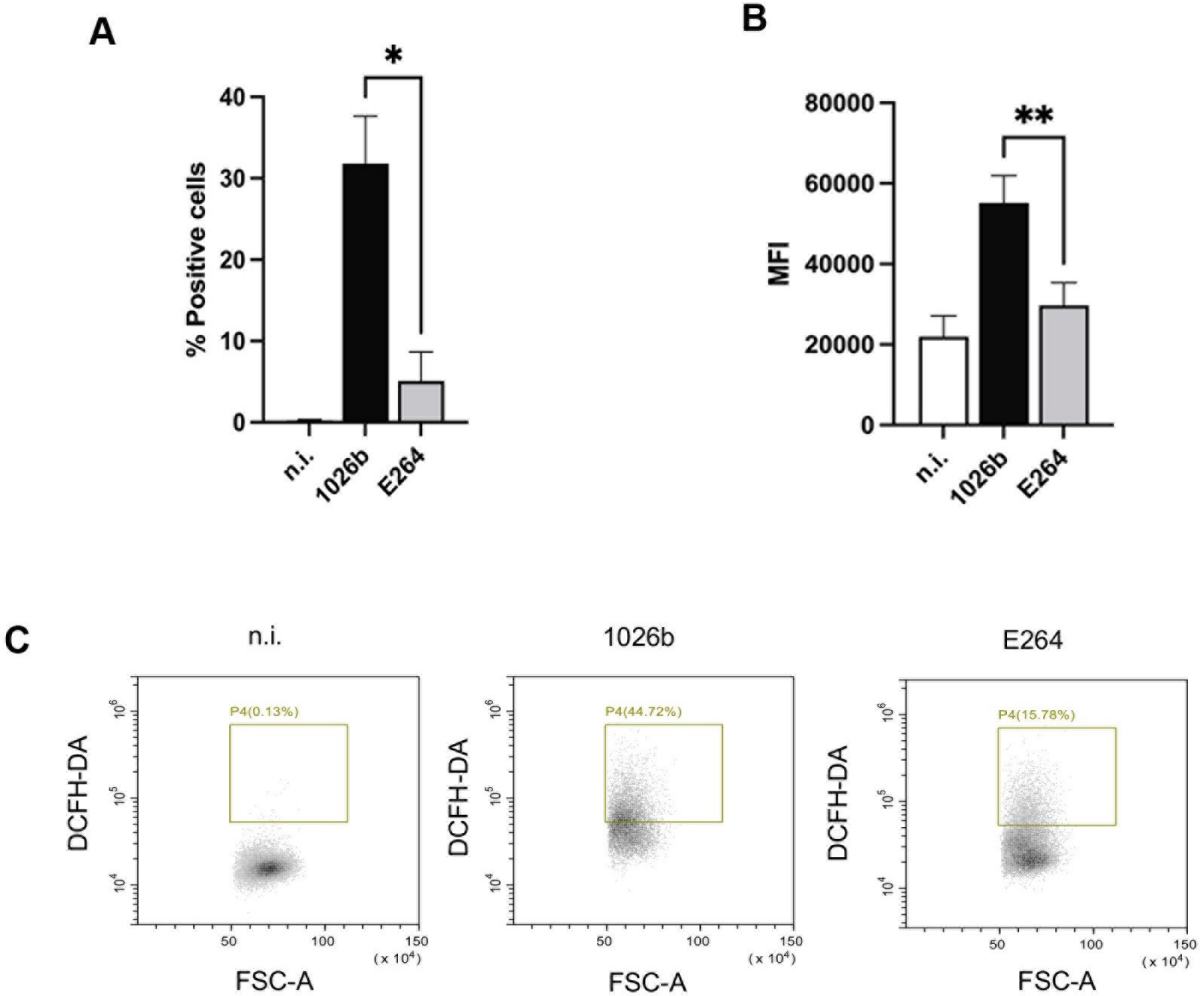
*B*. *pseudomallei*, but not *B*. *thailandensis* is able to produce ROS. Primary human monocytes were infected with *B*. *pseudomallei* or *B*. *thailandensis* at MOI of 10. After 30 min post infection, the levels of ROS production were determined by staining cells with 2’,7’-DCFH-DA (10 μM). The percentage of fluorescence-positive cells **(A)** and the mean fluorescence intensity (MFI) **(B)** were quantitated by using flow cytometry. Dot plot analysis of ROS production was shown from one representative out of three independent experiments **(C).** The data represent the results from at least 3 independent experiments from different donors. Data are represented as the mean± SEM. (*p < 0.05 and **p < 0.01). n.i. (not infected).

ROS plays a central role in inflammasome activation, leading to pyroptosis [28]. To investigate whether *Burkholderia* species could produce ROS in primary human monocytes, the cells were infected with *B*. *pseudomallei* and *B*. *thailandensis* and stained with DCFH-DA (ROS-specific fluorescent dye) before measuring ROS production. As revealed in Fig 5A–5C, *B*. *pseudomallei*-infected cells resulted in a significantly higher percentage of ROS positive cells and mean fluorescence intensity (MFI) than that of *B*. *thailandensis*-infected cells. These results indicated that only *B*. *pseudomallei* could produce significantly higher ROS in primary human monocytes during infection.

### ROS inhibition alters bacterial intracellular survival and pyroptosis in *B*. *pseudomallei*-infected primary human monocytes

**Fig 6:**
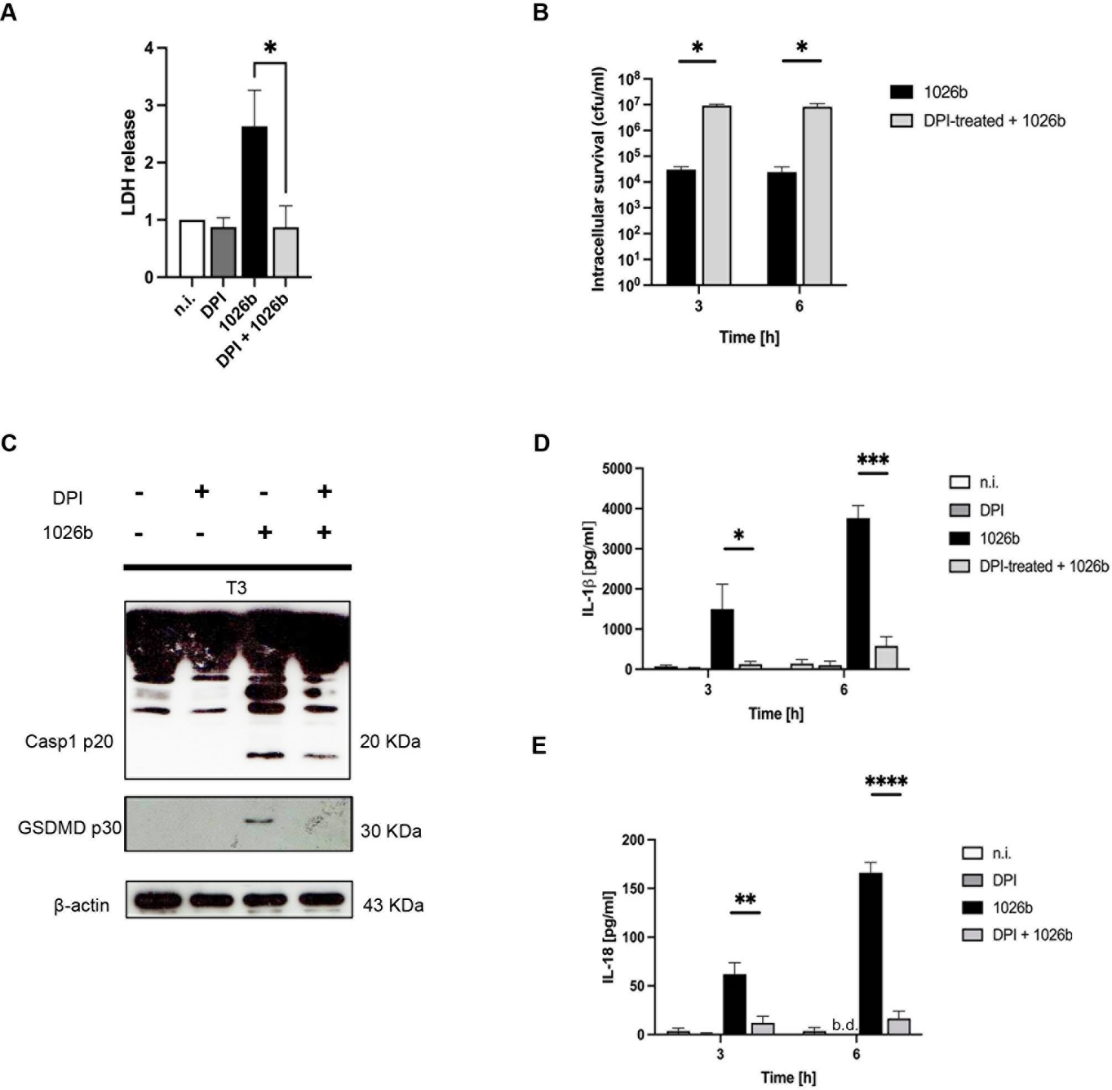
Pyroptosis in *B*. *pseudomallei*-infected primary human monocytes is mediated by ROS production. Primary human monocytes were pre-treated with 20 μM diphenyleneiodonium (DPI), an NADPH oxidase inhibitor, for 30 min before being infected with *B*. *pseudomallei* at MOI of 10 for 3 h. The cell culture supernatants of infected cells were collected at indicated time points. The LDH release was determined by cytotoxicity assay **(A)**. The intracellular survival was determined by the drop plate technique **(B)**. Immunoblot analysis was performed on cell lysates (3 h p.i.) **(C)**. IL-1β **(D)** and IL-18 **(E)** secretion were determined. The data represented the results of at least 3 independent experiments with different donors. Data are represented as the mean ± SEM. (*p < 0.05, **p < 0.01, ***p < 0.001 and ****p < 0.0001). n.i. (not infected), b.d. (below detection).

Next, we extended the study to investigate whether ROS production observed in *B*. *pseudomallei*-infected primary human monocytes related to pyroptosis activation and intracellular bacterial replication. To examine the role of ROS in pyroptosis activation, LDH levels in *B*. *pseudomallei*-infected primary human monocytes in the presence of ROS inhibitor (DPI) were analyzed. The data in Fig 6A demonstrated that inhibition of ROS led to a significant decrease in LDH release in *B*. *pseudomallei*-infected primary human monocytes. Additionally, in the presence of DPI, *B*. *pseudomallei*’s intracellular survival in infected primary human monocytes was significantly increased [Fig 6B]. Furthermore, the study investigated the effect of ROS production on the activation of caspase-1 and GSDMD in primary human monocytes. Inhibition of ROS by DPI in *B*. *pseudomallei*-infected primary human monocytes led to the attenuation of caspase-1 and GSDMD activation [Fig 6C]. This coincides with a decrease in IL-1β and IL-18 secretion during *B*. *pseudomallei* infection [Fig 6D and 6E]. These findings suggested that the generation of ROS is essential for the induction of pyroptosis as well as the control of intracellular survival of *B*. *pseudomallei* in primary human monocytes.

## Discussion

Melioidosis is an emerging life-threatening infectious disease caused by the Gram-negative intracellular bacterium, *B*. *pseudomallei*. It often presents with varied clinical manifestations and has a high fatality rate if misdiagnosed or left untreated [1]. In more than 50% of patients, the disease manifests as sepsis syndrome, which is a life-threatening condition that results in systemic inflammation and immune response and can lead to multiple organ dysfunction [2]. In contrast, the closely related species, *B*. *thailandensis* has occasionally been reported to cause pneumonia and sepsis in humans [29]. *B*. *thailandensis* is less virulent than *B*. *pseudomallei*, judging by LD50 and pathogenesis, but the behavior of these two species, such as the ability to multiply inside the cells, does not significantly differ, such as mouse macrophage cell line (J774A.1) and human monocyte-derive dendritic cells (MoDCs) [30–32]. Therefore, several groups of researchers used *B*. *thailandensis* as a model organism for studying *B*. *pseudomallei* [30,33]. However, *B*. *thailandensis* is not a good surrogate for *B*. *pseudomallei*. For example, the essential virulence factors such as polysaccharide capsules and lipid A species acylated with C_14:0_(2-OH) fatty acid are detected in *B*. *pseudomallei* but are not found in *B*. *thailandensis* [34].

Although it is now well established that *B*. *pseudomallei* can be genetically distinguished from *B*. *thailandensis*, the mechanisms whereby only the virulent *B*. *pseudomallei* can cause sepsis in acute melioidosis are still poorly understood [32,35]. In the present study, the virulent *B*. *pseudomallei* was compared to the non-virulent *B*. *thailandensis* during infection of primary human monocytes and THP-1 cells. Interestingly, both species of *Burkholderia* exhibit the same ability to invade primary human monocytes and THP-1 cells but fail to multiply after 6 h of infection [Fig 1]. Surprisingly, we found that the secretion of IL-1β and IL-18 was significantly higher only in primary human monocytes and THP-1 cells infected with *B*. *pseudomallei* as compared to those infected with *B*. *thailandensis* [Fig 3]. However, the levels of other cytokines, namely, IL-1RA, IL-10, IL-12p70, IL-15, and IL-23 were not significantly different between the two groups, suggesting that IL-1β and IL-18 may play a crucial role in the pathogenesis of *B*. *pseudomallei* infection [Fig 4].

In septic melioidosis, IL-1β and IL-18 levels are predominantly increased in the bloodstream of the patients [12,13]. The increased levels of these cytokines can also affect various cell types in the patient’s bloodstream. In general, IL-1β production has been reported to play several roles in various types of cells in response to bacterial infections, inducing other cytokines and stimulating specific types of adaptive immunity such as Th17 production. This can drive autoinflammation and increased expression of IFN-γ, GM-CSF, IL-22, and IL-23R with diminished expression of IL-10 [36,37]. Moreover, IL-18 may also promote the secretion of other pro-inflammatory cytokines such as TNF-α, IL-1β, IL-8, and GM-CSF, which leads to an increase in the expansion, migration, and activation of neutrophils during infections [38]. Therefore, the elevation of IL-1β and IL-18 secretion by infected monocytes may be the keys cytokines participating in sepsis by affecting other immune cells to orchestrate the secretion of other pro-inflammatory cytokine production, resulting in the cytokine storm as observed in acute melioidosis patients [39]. In addition, high levels of IL-1β production have also been reported to exert a harmful effect on the host during pulmonary infection of *B*. *pseudomallei* and drive severe Group A *Streptococcus* (GAS) disease, which could lead to organ injury [12,40,41].

Both IL-1β and IL-18 play a crucial role in the body’s innate immune defense against pathogens and are the key markers of pyroptosis. Although *B*. *pseudomallei*-induced cell death through pyroptosis has been demonstrated in several cell types, including primary human macrophages, only limited information of pyroptosis in human monocytes was demonstrated [24]. Our data showed that the infection of *B*. *pseudomallei* in both primary human monocytes and THP-1 cells resulted in a significant increase in LDH release compared to that of *B*. *thailandensis* infection. In order to release the content of cells, including IL-1β and IL-18, from the infected cells, caspase-1/4/5 activation is required to activate gasdermin D (GSDMD), leading to cell swelling and the formation of membrane pores [14]. In the present study, we also showed that activation of caspase-1 and the cleavage of GSDMD were consistently observed in *B*. *pseudomallei* infection but not in *B*. *thailandensis* infection [Fig 2]. This suggested that *B*. *pseudomallei* is not only a more potent inducer of pyroptosis than *B*. *thailandensis*, but pyroptosis might be a part of sepsis in melioidosis patients.

ROS is well established and plays a pivotal role in contributing to the early control of infections [42,43]. This radical can directly damage the DNAs and proteins of the pathogens, leading to intracellular killing [44]. The role of ROS in *B*. *pseudomallei* infection has been demonstrated in many cells, particularly human macrophages [24]. However, the role of ROS in monocytes infected with *B*. *pseudomallei* has not been reported. In the present study, we demonstrated that only *B*. *pseudomallei*, not *B*. *thailandensis* was able to produce high levels of ROS during infection in primary human monocytes. Additionally, in the presence of ROS inhibitor (DPI), the number of intracellular bacteria was significantly increased [Fig 6], suggesting that ROS also plays an important role in controlling intracellular *B*. *pseudomallei* replication in human monocytes. Previously, our group demonstrated earlier that ROS can also regulate pyroptosis in a macrophage cell line (Raw264.7) that is activated with CpG-ODN 1826 (a ligand of TLR9). Inhibition of ROS production in activated-Raw264.7 cells resulted in the attenuation of pyroptosis as judged by the decrease in LDH release, and caspase-11 and GSDMD activation [45]. Consistent with our study, in the presence of ROS inhibitor, the levels of LDH release and activation of caspase-1, GSDMD, as well as IL-1β and IL-18 secretion were suppressed in *B*. *pseudomallei*-infected monocytes [Fig 6]. Considering that pyroptosis is well established to involve in the intracellular killing of bacteria, therefore, ROS produced in *B*. *pseudomallei*-infected human monocytes may also indirectly contribute to bacterial intracellular killing via pyroptosis induction.

Numerous studies have highlighted the distinction between *B*. *pseudomallei* and *B*. *thailandensis* in various aspects [7,32,34]. Although these two *Burkholderia* strains are genetically similar, several virulence factors, such as T3SS or lipid A, are different between these two strains [34,46,47]. These variations may result in differences in human monocyte response. In the present study, we demonstrated that only *B*. *pseudomallei* could induce high levels of IL-1β and IL-18 during primary human monocyte infection. Since both of these cytokines can influence other blood immune cells to secret other proinflammatory cytokines, for example, TNF-α, and IFN-γ. Therefore, the elevation of IL-1β and IL-18 secretion by infected monocytes may contribute, at least in part, to sepsis observed in melioidosis patients.

Sepsis, caused by a cytokine storm, is one of the key factors for the high mortality rate in acute melioidosis caused only by *B*. *pseudomallei* infection. Notably, our current research provides possible novel evidence that pyroptosis induced by virulent *B*. *pseudomallei* may play a pivotal role in sepsis. These findings may contribute to understanding the mechanisms underlying pathogenesis in acute melioidosis.

## Supporting information

S1 Table Percentage uptake of *B*. *pseudomallei* and *B*. *thailandensis* in primary human monocytes and THP-1 cells. (PDF)

S1 Data Excel spreadsheet containing separate sheets that represent the values used to build the graphs and perform statistical analysis for Figs 1, 2, 3, 4, 5, and 6. (XLSX)
