# Supplementary material for: Differentiation in pyroptosis induction by Burkholderia pseudomallei and Burkholderia thailandensis in primary human monocytes, a possible cause of sepsis in acute melioidosis patients
Source: PLoS Negl Trop Dis. 2024 Jul 23;18(7):e0012368. doi: 10.1371/journal.pntd.0012368 (PMC11296640; doi:10.1371/journal.pntd.0012368)
Supplement: S1 Table — (PDF) [file pntd.0012368.s001.pdf]

% Uptake of *B. pseudomallei* and *B. thailandensis* in primary human monocytes and THP-1 cells

Primary human monocytes

Bacteria (strain)	% Uptake ($x10^{-1}$)
B. pseudomallei 1026b	3.2
B. thailandensis E264	3.5

THP-1 cells

Bacteria (strain)	% Uptake
B. pseudomallei 1026b	8.65
B. thailandensis E264	5.46

Uptake (%) = (Number of intracellular bacteria at 3h post-infection/Number of CFU added)x100; results represent means from at least three independent experiments, each performed in duplicate.
